# Value of Perfusion CT, Transcranial Doppler Sonography, and Neurological Examination to Detect Delayed Vasospasm after Aneurysmal Subarachnoid Hemorrhage

**DOI:** 10.1155/2012/231206

**Published:** 2012-09-24

**Authors:** Ekkehard Kunze, Mirko Pham, Furat Raslan, Christian Stetter, Jin-Yul Lee, Laszlo Solymosi, Ralf-Ingo Ernestus, Giles Hamilton Vince, Thomas Westermaier

**Affiliations:** ^1^Department of Neurosurgery, University of Wuerzburg, Josef-Schneider-Str. 11, 97080 Wuerzburg, Germany; ^2^Department of Neuroradiology, University of Heidelberg, Im Neuenheimer Feld 400, 69120 Heidelberg, Germany; ^3^Department of Neuroradiology, Universitt Würzburg, Josef-Schneider-Str. 11, 97080 Wuerzburg, Germany

## Abstract

*Background*. If detected in time, delayed cerebral vasospasm after aneurysmal subarachnoid hemorrhage (SAH) may be treated by balloon angioplasty or chemical vasospasmolysis in order to enhance cerebral blood flow (CBF) and protect the brain from ischemic damage. This study was conceived to compare the diagnostic accuracy of detailed neurological examination, Transcranial Doppler Sonography (TCD), and Perfusion-CT (PCT) to detect angiographic vasospasm. *Methods*. The sensitivity, specificity, positive and negative predictive values of delayed ischemic neurological deterioration (DIND), pathological findings on PCT-maps, and accelerations of the mean flow velocity (MVF) were calculated. *Results*. The accuracy of DIND to predict angiographic vasospasm was 0.88. An acceleration of MFV in TCD (>140 cm/s) had an accuracy of 0.64, positive PCT-findings of 0.69 with a higher sensitivity, and negative predictive value than TCD. *Interpretation*. Neurological assessment at close intervals is the most sensitive and specific parameter for cerebral vasospasm. PCT has a higher accuracy, sensitivity and negative predictive value than TCD. If detailed neurological evaluation is possible, it should be the leading parameter in the management and treatment decisions. If patients are not amenable to detailed neurological examination, PCT at regular intervals is a helpful tool to diagnose secondary vasospasm after aneurysmal SAH.

## 1. Introduction

Among other variables, cerebral infarction and symptomatic vasospasm are the most important postoperative risk factors for poor outcome after aneurysmal SAH [1] Both are consequences of a decreasing and insufficient brain perfusion that eventually causes a loss of neurological function and, finally, structural damage of brain tissue [2]. A variety of measures is undertaken to enhance cerebral blood flow (CBF) in SAH-patients developing delayed cerebral vasospasm (DCV). These include hyperdynamic therapy, intra-arterial and intrathecal drug infusion, intra-aortic balloon counterpulsation, and new experimental methods [3]. Endovascular treatment of DCV—balloon angioplasty and chemical vasospasmolysis—has proven to effectively enhance CBF and has been increasingly used for treatment of DCV in the last years [4]. The application of these modalities or their combination may be very helpful if treatment is started in time. However, the routine use of repeated four-vessel angiography is not justifiable because of high-radiation exposure. Therefore, other monitoring tools have to indicate upcoming vasospasm after aneurysmal SAH. If DCV is missed or treatment started too late, cerebral infarction might develop in spite of maximum treatment. A number of monitoring methods are available. The use of continuous invasive brain monitoring like bedside microdialysis or intracerebral measurement of tissue oxygenation or regional cerebral blood flow (rCBF) is discussed with controversy. The ideal monitoring is noninvasive, can be repeated any time without extensive technical setup, has a high-diagnostic accuracy, and gives information about the development of DCV and the decrease of cerebral perfusion early enough to start an adequate therapy and prevent cerebral infarction. Neurological assessment and TCD are noninvasive procedures. PCT obtained at regular intervals is only little invasive regarding the exposure to radiation. This study was designed to evaluate and compare these diagnostic modalities for their prognostic value to detect angiographic vasospasm and perform endovascular treatment.

## 2. Materials and Methods

The study was approved by the local ethics committee. The data was collected in the framework of a clinical trial assessing the effectiveness of magnesium treatment in aneurysmal SAH [5]. The patients analyzed in the present study resemble the control group of this clinical trial. In order to prevent a possible contamination of results by a drug with vasodilatory and neuroprotective potential, patients receiving magnesium medication were not included in this analysis. Informed consent was obtained from the patient or a legal guardian.

### 2.1. Inclusion- and Exclusion-Criteria

Patients were eligible for inclusion if they had suffered aneurysmal SAH no longer than 96 hours ago. Patients were not included if they were under 18 years of age, if treatment was discontinued at the time of hospital admission due to a poor clinical state on admission, if there was a history of preceding aneurysmal SAH, or if therapy was scheduled to be continued elsewhere after obliteration of the aneurysm. Further exclusion criteria were pregnancy, cancer, atrioventricular block, preexisting neuromuscular disease, and renal failure. If serum creatinine values were above 133 *μ*mol/L (1.5 mg/100 mL), values were controlled 8 hours later after fluid substitution with 1.5 liters of Ringer's solution. If serum creatinine had declined below 133 *μ*mol/L, the patient was included in the study.

All patients were admitted to the neurosurgical intensive care unit. The intervals from the presumable aneurysm rupture until hospital admission were documented as well as comorbidity and medication. The neurological examination was performed according to an examination protocol which included assessment and documentation of the Glasgow Coma Score (GCS), deficits of awareness (person, place, time, situation), deficits of cranial nerves, visual field deficits, motor and sensory deficits of the upper and lower extremities, coordination deficits, and speech abnormalities. 

### 2.2. Assessment of Study Parameters

Neurological examination: Detailed neurological examination was conducted 3 times a day (1 a.m., 9 a.m., and 5 p.m.) by the neurosurgeon on duty on the ICU following the above mentioned neurological examination protocol. Any secondary deterioration of the neurological state was considered abnormal. If secondary deterioration was detected and confirmed by the senior neurosurgeon on duty, blood counts and serum electrolytes were determined and a CT-scan was obtained. Delayed Ischemic Neurological Deficit (DIND) was defined as a secondary neurological deterioration after exclusion of rebleeding or intracerebral hemorrhage, hydrocephalus, seizure, or electrolyte disturbance. The examiners were not involved in the conduction and analysis of Transcranial Doppler Sonography and Perfusion-CT.

Transcranial Doppler Sonography (TCD): The TCD examination was conducted daily after the neurological examination in the morning by a trained and experienced medical technician who was blinded to the result of the neurological examination. The mean flow velocities (MFV) in the vessel trunks of both middle, anterior, and posterior cerebral arteries (MCA, ACA, PCA) were determined via a temporal window, the MFV in the basilar artery was determined via the foramen magnum. Sonographic vasospasm was defined as a MFV >140 cm/s in the MCA, ACA and/or PCA or a MFV of >90 cm/s in the basilar artery.

Perfusion CT: Native CT and PCT were obtained at day 3 or 4, day 6 or 7, and day 9 or 10 after admission or at any other point if it was thought to be of diagnostic relevance. If patients were hospitalized longer, further CT/PCT-scans were obtained at 3-day intervals. For the determination of irreversible brain infarction on native CT, diagnostic criteria were applied as previously described [6]. For the native CT, slice thickness was 5 mm for the posterior fossa and 8 mm for the cerebrum (Somatom Plus 4 Volume Zoom, Siemens, Erlangen, Germany). Slice positioning was above the orbital roof in the supraorbitomeatal direction. 

**Figure 1:**
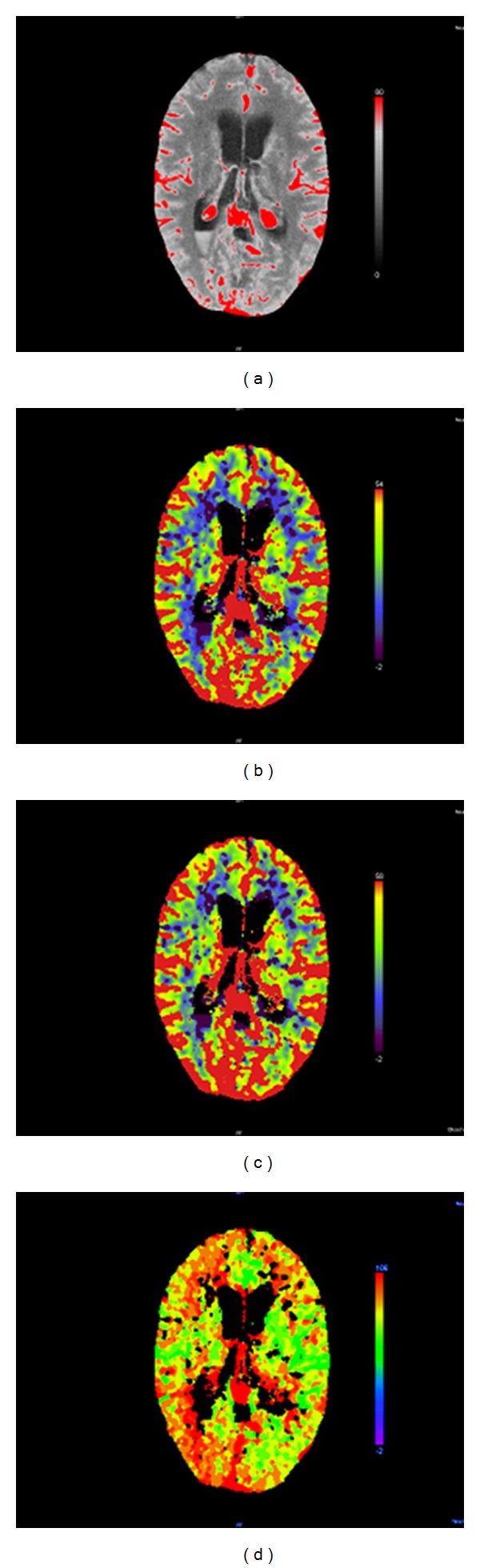
Perfusion-CT of a 40-year-old female patient with subarachnoid hemorrhage Hunt/Hess 3 caused by rupture of a right posterior communicating artery aneurysm. (a) Basis scan, (b) CBF map, (c) Blood Volume map, and (d) time to peak map. While the CBF map and Blood Volume map do not show signs of a perfusion deficit, the TTP map depicts an asymmetry in the posterior MCA-territory indicating spasm of the right terminal carotid artery and MCA.

Perfusion CT was obtained as previously described [7]. In brief, two adjacent 10-mm slices were positioned at the level of the basal ganglia with the same angulation as for native CT. A bolus of 50 mL of nonionic contrast medium (Imeron 400, Bracco, Konstanz, Germany) was administered by a power injector into a central venous catheter at a flow rate of 4 mL/s followed by 30 mL of saline. Four seconds after beginning of the bolus, 40 images were collected at each slice level at a rate of two images per second (120 kV, 110 mAs, matrix 512 × 512). For PCT analysis a commercially available software was used (Perfusion CT, Siemens). PCT color maps were qualitatively assessed using a visual grading scale [7, 8]. A positive visual assessment was noted for side-to-side asymmetries or clear bilateral defects suggesting a decrease in Cerebral Blood Flow (CBF), Cerebral Blood Volume (CBV), or an increase in Time To Peak (TTP) (Figure 1). CT and Perfusion-CT was analyzed by trained neuroradiologists (M.P., L.S.) and a trained neurosurgeon (C.S.) blinded to the patient data, neurological exam, and TCD values.

### 2.3. Target Parameters

The target parameter was angiographic vasospasm. Patients were forwarded to digital subtraction angiography (DSA) in case of neurological deterioration which was not explained by other reasons (rebleeding, seizure, and electrolyte disturbance), steep elevations of flow velocities in TCD, and/or clear and newly appearing deficits in perfusion CT. Angiographic vasospasm was defined as a narrowing of the diameter of a vessel trunk of more than 30% and a significant consecutive delay of contrast flow distal to the spastic segment. The DSA images were digitally stored, measured, and reanalyzed by a neuroradiologist blinded to the results of the neurological exam, TCD, and PCT. If the criteria for vasospasm were fulfilled and the spastic segment was accessible to a microcatheter, balloon angioplasty was performed. If the criteria were fulfilled but the segment was not accessible for a microcatheter, chemical spasmolysis with papaverin or nimodipine was performed.

Diagnostic accuracy of neurological assessment, TCD, and qualitative CTP was determined by sensitivity, specificity, and positive and negative predictive values (PPV, NPV). 

## 3. Results

The studies were conducted in the framework of a clinical trial assessing the therapeutical potential of magnesium sulfate to protect from secondary ischemic events after SAH [5]. In this study, magnesium showed a spasmoprotective effect. To prevent contamination of the data, only patients of the control group of this study are presented here. 

### 3.1. Patient Characteristics

53 patients completed the study protocol and were analyzed. Secondary cerebral infarctions occurred in 27 patients. 39 territorial and 11 lacunar infarctions were counted. 

### 3.2. Neurological Assessment

31 patients were amenable to neurological assessment through major parts of their hospitalization. 18 episodes of DIND were registered in 15 patients. 11 times, patients were forwarded to DSA on the same day or on the next day. In 9 of these patients, DSA showed significant vasospasm and all were treated by balloon dilatation. 5 times, DSA was performed for other reasons than DIND and was negative in all 5 cases (sensitivity for DIND to predict angiographic vasospasm 1.00, specificity 0.33, PPV 0.69, NPV 1.00).

### 3.3. Transcranial Doppler Sonography (TCD)

MFV in the large intracranial vessel trunks was assessed daily by TCD in all patients from admission until the end of the treatment. Cerebral vasospasm in TCD was defined as MFV higher than 140 cm/s. 45 patients had MFV of more than 140 cm/s for 1 day or longer during the hospital stay. 

50 angiographies for suspected vasospasm were performed in 29 patients. 37 of these procedures were paralleled by MFV over 140 cm/s in one or more intracranial vessel trunks. 27 of these procedures revealed significant vasospasm and were treated by balloon angioplasty or chemical vasospasmolysis. 13 times, MFV was lower than 140 cm/s and angiography was performed for other reasons (DIND, PCT). Of these patients, 8 showed angiographic vasospasm (sensitivity for accelerated flow velocity >140 cm/s to predict angiographic vasospasm 0.77, specificity 0.44, PPV 0.73, and NPV 0.62).

### 3.4. Perfusion CT (PCT)

Serial CT-scans and PCT-maps were obtained on admission, on days 3/4, 6/7, 9/10, or any other day when it was considered to be of diagnostic relevance. 92 CBF- and Blood Volume maps were positive. 27 positive examinations were followed by angiography. 19 of these angiographies showed significant vasospasm and 8 were negative. In 19 cases, angiography was performed for other reasons. 8 were negative and 12 showed significant angiographic vasospasm requiring treatment (sensitivity for CBF- and Blood Volume maps to predict angiographic vasospasm 0.61, specificity 0.47, PPV 0.70, and NPV 0.37). Except for 2 cases, CBF- and blood volume maps were positive when infarction or hemorrhage or postoperative contusion was already demarcated in plain CT-scans. 

97 TTP-maps were positive and were followed by angiography 38 times. 27 of these angiograms showed significant vasospasm and 11 were negative. In 6 patients, angiography was performed for other reasons. 4 of these angiograms were negative, 2 were positive (sensitivity for TTP maps to predict vasospasm 0.93, specificity 0.27, PPV 0.71, and NPV 0.67). 

### 3.5. Simultaneous Positive Findings in TCD and Time To Peak

In 45 patients, both TCD-values and TTP-maps were obtained prior to angiography. Angiography showed a positive result in 30 cases. 23 of these patients had both MFV higher than 140 cm/s and positive TTP maps. 14 angiographies were negative. 6 of these patients had both elevated MFV in TCD and positive TTP maps (sensitivity for both positive TCD- and TTP-findings at the same time to predict angiographic vasospasm 0.74, specificity 0.57, PPV 0.79, and NPV 0.53).

## 4. Discussion

This study assessed neurological examination, Transcranial Doppler Sonography, and Perfusion CT for their ability to predict cerebral vasospasm after aneurysmal SAH. DSA is the gold standard in the diagnosis of cerebral vasospasm and other cerebrovascular pathologies. [9, 10]. Magnetic resonance angiography (MRA) has been reported to exaggerate vascular narrowing [11]. CT-angiography (CTA) has shown good accuracy to detect vasospasm in the basal vessel trunks but less sensitivity for peripheral vasospasms [9]. One major advantage of DSA is the possibility to perform balloon dilatation and/or pharmacological angioplasty immediately after the diagnostic procedure. However, the administration of contrast medium, the invasiveness of the procedure, and the exposure to radiation prohibit its serial use for diagnostic purposes. Therefore, routine DSA is not implemented in our treatment protocol for SAH patients. 

It may be discussed what extent of vessel narrowing should be regarded vasospasm. Many authors define severe vasospasm as the narrowing of vessel diameter of more than 50% [12, 13]. Many patients who develop delayed cerebral ischemia (DCI), however, have segmental arterial narrowing of less than 50% and even patients with no marked arterial narrowing can develop DCI [14]. Dankbaar et al. found that 57% of patients with angiographic narrowing of more than 50% and 37% of patients with arterial narrowing of 25–50% developed DCI [15]. A segmental narrowing of the vessel diameter of 30% equals a reduction of cross-section to 49% and a decrease of flow volume to 24% according to the Hagen-Poiseuille equation. This has to be compensated by an at least twofold increase of flow velocity in order to ascertain cerebral perfusion at levels above the ischemic thresholds [2]. Therefore, our definition of angiographic vasospasm as well as for its endovascular treatment was arterial narrowing of more than 30% and a decreased circulation time in the respective part of the cerebral vasculature. 

A number of invasive methods like microdialysis and intracerebral CBF-measurement have been tested for their value to detect DCV in patients after aneurysmal SAH. Those procedures bear the risks of placement and long-term monitoring with intracerebral probes like infection and probe dislocation. Furthermore, they are only successful if the probes have been placed into an area if the brain which will eventually be affected by decreased perfusion. This cannot be reliably predicted. Therefore, these methods have a higher risk for false negative findings. It was the aim of the present study to examine noninvasive diagnostic measures for their diagnostic accuracy. The choice of the appropriate target parameter is debatable. Classically, cerebral infarction and the appearance of DIND as expressions of progressive ischemia are used as target parameters to assess cerebral ischemic events [16]. To use angiographic findings as the study end point, however, is a therapeutic approach. Endovascular balloon angioplasty of narrowed vessel trunks and chemical vasospasmolysis are known to significantly raise CBF above ischemic values and are, therefore, prophylactic measures to prevent ischemia and permanent neurological deficit [4, 17, 18]. 

Neurological examination has been performed using a standardized protocol assessing awareness, psychomotor function, cranial nerves, and motor and sensory function. Patients that were amenable to this detailed examination were tested following this protocol at least 3 times a day. Although the number of patients is small, out results show that neurological assessment has an outstanding diagnostic accuracy. Of all calculated values, the high-negative-predictive value is the most important parameter as it excludes that a patient will not be transferred to adequate treatment if the diagnostic finding is negative. However, a protocol as detailed as in the present study is not practicable in somnolent or comatose patients. In these patients, other diagnostic procedures have to be employed which can be performed independently of the state of consciousness. TCD and PCT are diagnostic tools that are readily available, easy to performm and noninvasive. TCD can be repeated at any time, PCT can be obtained subsequent to native CT when the latter are conducted at regular intervals in comatose SAH patients to exclude secondary infarction, rebleeding, edema, and so forth. 

Both methods have their limitations and are influenced by factors other than the narrowing of brain supplying blood vessels. TCD evaluates flow velocities in intracranial vessels. The measurement of TCD in the anterior circulation depends on the patient's “ultrasound window”, a part of the temporal bone which has to be permeable for ultrasound waves [19, 20]. TCD measurements are strongly examiner-dependent. To exclude major interobserver variability, TCD measurements were obtained by a trained medical assistant in our study. Furthermore, flow velocities not only increase and decrease with changes of the vessel diameter but also with changes in cardiac output, blood pressure and blood viscosity. Therefore, a variety of factors, such as hyperdynamic therapy can contribute to elevations of flow velocities. Finally, TCD can detect large vessel spasms but not peripheral vasospasms.

Color-coded CTP-maps are gathered from serial CT-slices which are repeated during the infusion of contrast medium. Typically, they are positioned relatively close to the skull base an can miss perfusion deficits in more apical areas of the brain, in territories supplied by smaller branches of the major vessel trunks. PCT-findings can be contaminated by infarcted tissue, postoperative contusion, and intracerebral hemorrhage. The acquisition of PCT maps is, as well, dependent on the examiner. However, PCT maps are color-coded and detection of asymmetries or bilateral defects compared to other vessel territory is rather reliable and of acceptable interobserver variability [7] Wintermark et al. proposed a user-friendly visual grading system which was used in this study [8]. The authors found that the Mean Transit Time (MTT), a parameter similar to the Time to Peak obtained in our study had the highest accuracy to predict angiographic vasospasm [8]. Turowski and coworkers found that a prolonged MTT correlates with clinical deterioration and angiographic vasospasm [21]. CBF- and Blood-volume-maps had lower accuracy to predict angiographic vasospasm. 

In the present analysis, we found that TTP maps had a higher sensitivity and a higher NPV than TCD to predict angiographic vasospasm. Blood volume and CBF-analysis, however, seem to be of less importance. Except for 2 cases, Blood Volume and CBF maps only showed pathological findings when infarction was already demarcated in plain CT-scans. The combined analysis of TCD and TTP maps increases specificity, however, at the expense of a lower NPV. 

The present analysis can only show a trend and compares different noninvasive methods to predict angiographic vasospasm which might be treated by balloon angioplasty or chemical vasospasmolysis. The indication for angiography did not follow a strict algorithm. Clinical deterioration that did not timely resolve upon enforced hyperdynamic therapy contributed to the decision as well as high elevations or sudden increases of flow velocities in TCD and pathological findings in PCT. In many cases, both TCD- and TTP-findings were pathological at the same time. Therefore, there will be a rather large number of undiagnosed cases, in particular concerning patients with negative clinical, TCD-, or PCT-findings and the number of true negative findings is likely to be highly underestimated. Above all, this will result in an incorrectly low specificity. Regarding specificity, therefore, the present study, does not claim to supply valid absolute values. In a previous study evaluating the prognostic value of TCD and PCT to predict delayed cerebral infarction [7], both diagnostic methods showed a sensitivity comparable to the results presented here but a much higher specificity (TCD 0.5, TTP 0.67). In contrast to the present series, the primary end point of that study was delayed infarction on native CT-scans which were obtained at regular intervals and, thus, supply valid information about the eventual outcome of negative diagnostic findings. Absolutely reliable prognostic values to predict angiographic vasospasm could only be determined if each clinical examination, TCD- and PCT-examination—positive or negative—was followed by angiography, which is neither practicable nor responsible.

## 5. Conclusion

The present data shows a superior diagnostic value of detailed neurological evaluation. Neurological assessability should, therefore, be maintained if ever possible and quickly reestablished after procedures for which patients need anesthesia, for example, endovascular and operative procedures. If patients are comatose and not amenable to a reliable neurological evaluation or have to be kept under analgosedation due to elevated intracranial pressure, technical tools have to be used to diagnose cerebral vasospasm. In these cases, repeatedly obtained PCT is a valuable screening tool to detect vasospasm and to decide whether a patient should be forwarded to angiography.
